# Differences in MTHFR and LRRK2 variant’s association with sporadic Parkinson’s disease in Mexican Mestizos correlated to Native American ancestry

**DOI:** 10.1038/s41531-021-00157-y

**Published:** 2021-02-11

**Authors:** Elizabeth Romero-Gutiérrez, Paola Vázquez-Cárdenas, Hortensia Moreno-Macías, José Salas-Pacheco, Teresa Tusié-Luna, Oscar Arias-Carrión

**Affiliations:** 1 Unidad de Trastornos del Movimiento y Sueño, Hospital General Dr. Manuel Gea González, Ciudad de México, México; 2 Programa de Doctorado en Ciencias Biomédicas, Universidad Nacional Autónoma de México, Ciudad de México, México; 3 Centro de Innovación Médica Aplicada, Hospital General Dr. Manuel Gea González, Ciudad de México, México; 4 Departamento de Economía, Universidad Autónoma Metropolitana, Ciudad de México, México; 5 Instituto de Investigación Científica, Universidad Juárez del Estado de Durango, Durango, México; 6 Unidad de Biología Molecular y Medicina Genómica, Instituto Nacional de Ciencias Médicas y Nutrición Salvador Zubirán, Ciudad de México, México; 7 Departamento de Medicina Genómica y Toxicología Ambiental, Instituto de Investigaciones Biomédicas, UNAM, Ciudad de México, México

**Keywords:** Parkinson's disease, Risk factors, Predictive markers

## Abstract

Parkinson’s disease (PD), a common neurodegenerative disorder, has a complex etiology where environmental and genetic factors intervene. While a number of genes and variants have been identified in recent decades as causative or protective agents of this condition, a limited number of studies have been conducted in mixed populations, such as Mexican Mestizos. The historical convergence of two founding groups and three ethnicities, and the increasing north-to-south gradient of Native American ancestry in Mexico resulted in a subpopulation structure with considerable genetic diversity. In this work, we investigate the influence of 21 known susceptibility variants for PD. Our case–control study, with a cohort of 311 Mexican Mestizo subjects, found a significant risk association for the variant rs1491942 in *LRRK2*. However, when stratification by ancestry was performed, a risk effect for *MTHFR* rs1801133 was observed only in the group with the highest percentage of European ancestry, and the PD risk effect for *LRRK2* rs1491942 was significant in subjects with a higher ratio of Native American ancestry. Meta-analyses of these SNP revealed the effect of *LRRK2* rs1491942 to be even more significant than previously described in populations of European descent. Although corroboration is necessary, our findings suggest that polymorphism rs1491942 may be useful as a risk marker of PD in Mexican Mestizos with greater Native American ancestry. The absence of associations with the remaining known risk factors is, in itself, a relevant finding and invites further research into the shared risk factors’ role in the pathophysiological mechanisms of this neurodegenerative disorder.

## Introduction

Parkinson’s disease (PD), a common progressive and incurable neurodegenerative disorder, especially prevalent among the elderly, is estimated to affect >6 million people worldwide^1–3^. Clinical manifestations include motor symptoms, such as bradykinesia, resting tremor, rigidity, and deterioration of postural reflexes. In addition, non-motor alterations, e.g., sleep disorders, autonomic dysfunction, and cognitive impairment, adversely affect the quality of life, cause disability, or even mortality. Although the etiology of PD is complex, evidence suggests it is caused by the interaction of environmental and genetic factors^1,3^.

Studies conducted in recent decades have identified a number of genes and variants associated with PD^4–12^. It is estimated that 5–10% of all PD cases have a genetic etiology linked to forms with monogenic Mendelian inheritance patterns. These forms are attributed to various loci containing genes, such as *SNCA*, *PRKN*, *PARK7*, and *LRRK2* (refs. ^13–15^). In the rest of the cases, called sporadic, genetic susceptibility factors have also been demonstrated. So far, >90 risk loci have been identified; the associated variants are mainly single-nucleotide polymorphisms (SNPs)^5,8,10,12,14^. Some of these SNPs are located within or very close to loci linked to the familial forms mentioned above, which indicates that changes in the sequence of these genes are likely to be implicated in the key biological processes of PD development^14,16^. The identification and functional characterization of these genetic changes have provided information on the cellular and subcellular mechanisms contributing to PD-related neurodegeneration^15–21^.

Despite advances in the typification of PD’s genetic susceptibility factors, interpretation of these findings is still controversial. This limitation is evidenced in a GWAS study by Foo et al.^12^, that investigated PD risk loci in an Asian cohort and then compared the results with those of European populations. Although they report substantial overlap in genetic risk factors, the similarities between the two groups are incomplete. In addition to these reported differences is the bias in information from ethnically diverse groups due to the scarcity of genomic data from populations other than Caucasians and Asians in current studies^20,21^. It is important to consider this bias as the differences in demographic histories and adaptation processes endured by different populations are likely to have influenced the genetic architecture of complex diseases such as PD in these groups.

The demographic history of a given population is one of the contributing factors to the impact genetic changes have on the incidence of PD in that population. When the number of individuals that gave rise to a population is limited, there is likely to be a representative bias of some of their alleles in the following generations. Migration processes, mutations, selective pressures, and genetic drift can contribute in a determinant way to the presence and frequency of allelic variations^22–25^. These changes are especially prevalent in mixed populations^26^ like Mexico, where the majority of individuals are Mestizo, i.e., of Native American, European, and African ancestral origins^23,25,27^.

According to the most accepted hypothesis, American natives originated from East Asian groups that crossed the Bering Strait ~16 thousand years ago. Once in America, they expanded from the northern to the southern continent, in different settlements from Alaska to Chile. As these original groups inhabited the new environments, they underwent adaptive processes, selective pressures, long migrations, and isolation, which resulted in a reduction of genetic diversity in the population (bottlenecks and founder effect). For the Native Americans in Mexico, a decisive second event occurred with the European conquest and colonization. The arrival of Spaniards accompanied by African slaves caused a decrease in the number of Native American settlers, due, among other factors, to their susceptibility to new European diseases and wars. Over time, the miscegenation of Europeans, surviving Native Americans, and Africans took place. These historical characteristics are all reflected in the heterogeneous structure of the current Mexican population, which shows significant genetic diversity compared with other populations^24,28^.

To date, 22 articles have been published that address the genetics of PD in Mexican Mestizos^29–50^. These studies analyzed alterations in 17 genes (*SNCA, PINK1, PRKN, GBA, LRRK2, MTHFR, LRRK2, APOE, SYT11, DRD2, ANKK1, PARK7, MAPT, ALDH1, NR4A2, tRNAGln,* and *mtATP6*). Their results identify eight SNPs as potential risk factors for PD in Mexican subjects (rs385705916, rs356220, rs356203, rs7684318, and rs2736990 in the *SNCA* gene, rs421016 in the *GBA* gene, rs35479735 in the *NR4A2* gene, and rs1801133 in the *MTHFR* gene). When these findings were compared with the GWAS results from European and Asian populations, only one polymorphism (rs356203 in the SNCA gene) was found in common^10^. While this discrepancy may be due to insufficient statistical power, it could also be explained by genetic and environmental diversity among populations. In this work, we investigate the incidence of genetic variations that have previously been associated with PD in a Mexican Mestizo population. In addition, a novel panel of 32 Ancestry Informative Markers (AIMs)^51^ was used to estimate the gradient of European and Native American ancestry in our study subjects. This analysis of the subpopulation structure allowed us to assess PD risk association according to the percentage of Native American ancestry.

## Results

### Demographic and clinical characteristics

When comparing demographic and clinical characteristics between the groups, differences attributable to the place of recruitment are ruled out (Supplementary Table 1). The demographic and clinical characteristics of the 118 PD cases and 193 controls are summarized in Table 1. No differences are observed in age, sex, BMI, glucose levels, or cognitive deterioration; however, significant dissimilarities were found in total cholesterol and uric acid levels and frequency of depression. Total cholesterol levels were lower in PD cases at 175 mg/dl compared to controls at 195 mg/dl (*p* < 0.001). Similarly, uric acid levels were less in the cases (5.23 mg/dl) vs controls (6.035, *p* < 0.001). Also, the frequency of depression was higher in cases (72.32%) compared to controls (49.15%, *p* < 0.001). Case’s total Unified Parkinson’s Disease Rating Scale (UPDRS) scores were 72 ± 38, and UPDRS motor scores were 40 ± 23 with 2.5 ± 1 on the Hoehn and Yahr (HY) rating scale. The average age of PD onset was 64.08 ± 10.46 years; only 11 (9%) patients had an age of onset <50 years; in all cases, the subjects reported no family history of PD.

**Table 1:** Clinical and demographic characteristic of the study population.

	Cases (*n* = 118)	Controls (*n* = 193)	*p* Value
Males, *n*(%)	60(50.8)	97(50.2)	0.999^a^
Age at enrollment, years	69.92 ± 10.01	69.80 ± 8.63	0.914^b^
BMI, kg/m^2^	27.38 [19–38]	27.19 [19–49]	0.615^c^
Total cholesterol, mg/dl	175 [100–270]	195 [80–276]	0.0001^c^
Glucose, mg/dl	102 [67–217]	110.60 [74–234]	0.0181^c^
Uric acid, mg/dl	5.23 ± 1.60	6.035 ± 1.38	<0.0001^b^
Cognitive impairment, *n*(%) (by MMSE-test)	37(32.74%)	45(26.98%)	0.298^a^
Depression, *n*(%) (by HAM-D test)	81(72.32%)	87(49.15%)	<0.001^a^
Age at onset, years	64.08 ± 10.46		
Disease duration, years	5.93 ± 4.93		
UPDRS total score	72 ± 38		
UPDRS III score	40 ± 23		
HY scale	2.5 ± 1		

Shows mean values (Standard deviation or interquartile range) and frequency (%). Skewness and kurtosis tests were performed for normality.

*BMI* body mass index, *HAM-D* Hamilton Depression Rating Scale, *MMSE* Mini-Mental State Exam, *UPDRS* Unified Parkinson’s Disease Rating Scale: total score and score for Part III—Motor Examination, *HY* Hoehn and Yahr scale.

Reported *p* values were determined with a ^a^Fisher’s exact test, ^b^Student’s *t* test, or ^c^*U* Mann–Whitney.

### Genotypic characteristics

We investigated 21 SNPs as associated factors in PD; however, the polymorphisms rs947211, rs356220, and rs2736990 were discarded from further analysis due to their pattern of linkage disequilibrium. The polymorphisms rs34778348 and rs33949390 in the *LRRK2* gene were found to be monomorphic in our sample and were also discarded. Of the remaining 16 SNPs, a significant PD risk association was found for the allelic and genotype frequencies of the polymorphism rs1491942; all stated confidence intervals (CI) are 95%.

**Fig. 1:**
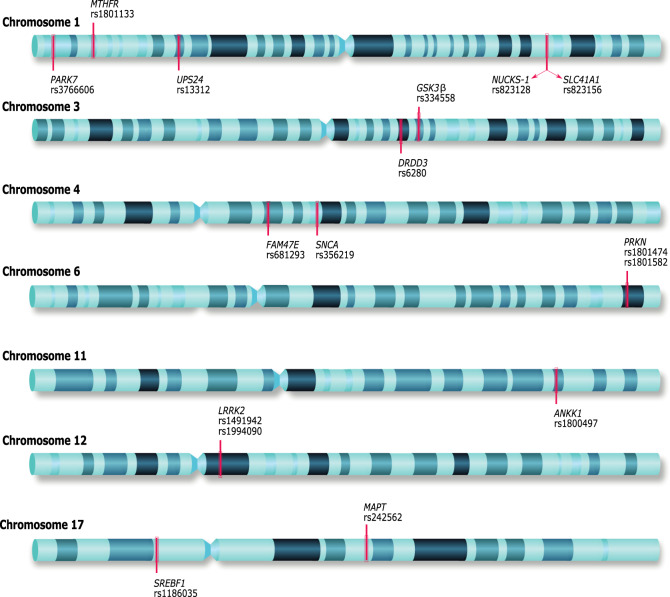
Chromosomal location of the 16 single-nucleotide polymorphisms selected for this research. Genes symbols and reference SNP numbers. Genes: PARK7 Parkinsonism associated deglycase, *MTHFR* Methylenetetrahydrofolate reductase; *USP24* Ubiquitin specific peptidase 24, *NUCKS1* Nuclear casein kinase and cyclin dependent kinase substrate *SLC41A1* Solute carrier family 41 member 1, *DRD3* Dopamine receptor D3, *GSK3B* Glycogen synthase kinase 3 beta, *FAM47E* family with sequence similarity 47 member E, *SNCA* Synuclein alpha, *PRKN* Parkin RBR E3 ubiquitin-protein ligase, *ANKK1* Ankyrin repeat, and kinase domain containing 1, *LRRK2* Leucine-rich repeat kinase 2, *SREBF1* sterol regulatory element-binding transcription factor 1 *MAPT* Microtubule-associated protein tau.

For SNP rs1491942 in the *LRRK2* gene, the estimated risk association under an additive model was odds ratio (OR) 1.71 [1.22–2.40] *p* 0.002. The selected SNPs’ genomic localization is shown in Fig. 1; no deviation from Hardy–Weinberg equilibrium (HWE) is observed in the control group. The allelic, genotyping distribution, and OR estimation of the SNPs are shown in Table 2.

**Table 2:** Allele and genotype frequencies in PD patients and controls.

Gene SNP	Group	MA *n* (freq)	*p*^allelic^	Genotype			*p*^genotype^	OR^a^	*p*^OR^
LRRK2 rs1491942	PD	155 (0.67)	0.003	CC	CG	GG	0.01	1.71 (1.22–2.40)	0.002
				14 (0.12)	53 (0.45)	51 (0.43)			
	CNT	205 (0.53)		44 (0.22)	93 (0.48)	56 (0.29)			
MTHFR rs1801133	PD	137 (0.58)	0.01	CC	CT	TT	0.041	1.54 (1.11–2.15)	0.01
				23 (0.19)	53 (0.45)	42 (0.36)			
	CNT	183 (0.47)		55 (0.29)	93 (0.48)	45 (0.23)			
USP24 rs13312	PD	20 (0.09)	0.99	CC	CG	GG	0.99	0.91 (0.49–1.71)	0.79
				96 (0.83)	18 (0.16)	1 (0.01)			
	CNT	24 (0.10)		104 (0.83)	20 (0.16)	2 (0.02)			
PARK7 rs3766606	PD	20 (0.03)	0.67	GG	GT	TT	0.83	0.87 (0.49–1.54)	0.64
				99 (0.84)	18 (0.15)	1 (0.09)			
	CNT	37 (0.06)		157 (0.81)	35 (0.18)	1 (0.05)			
NUCKS1 rs823128	PD	32 (0.13)	0.90	AA	AG	GG	0.56	0.94 (0.58–1.50)	0.80
				90 (0.76)	24 (0.20)	4 (0.04)			
	CNT	55 (0.14)		142 (0.74)	47 (0.24)	4 (0.02)			
SLC41A1 rs823156	PD	51 (0.22)	0.29	AA	AG	GG	0.09	0.79 (0.54–1.17)	0.25
				76 (0.64)	33 (0.28)	9 (0.08)			
	CNT	99 (0.26)		105 (0.54)	77 (0.40)	11 (0.06)			
GSK3B rs334558	PD	85 (0.36)	0.43	AA	GA	GG	0.44	1.14 (0.81–1.61)	0.42
				51 (0.43)	49 (0.41)	18 (0.15)			
	CNT	127 (0.33)		86 (0.45)	87 (0.47)	20 (0.10)			
DRD3 rs6280	PD	99 (0.42)	0.16	TT	TC	CC	0.23	0.75 (0.54–1.05)	0.10
				42 (0.36)	52 (0.44)	24 (0.20)			
	CNT	188 (0.49)		51 (0.26)	96 (0.49)	46 (0.24)			
FAM47E/ SCARB2 rs6812193	PD	49 (0.21)	0.4	CC	CT	TT	0.31	1.2 (0.80–1.82)	0.37
				78 (0.66)	31 (0.27)	9 (0.08)			
	CNT	69 (0.18)		131 (0.68)	55 (0.26)	7 (0.04)			
SNCA rs356219	PD	90 (0.38)	0.21	GG	AG	AA	0.29	0.79 (0.57–1.11)	0.18
				47 (0.40)	52 (0.44)	19 (0.16)			
	CNT	168 (0.44)		60 (0.31)	98 (0.51)	35 (0.18)			
PARK2 rs1801474	PD	36 (0.15)	0.48	CC	CT	TT	0.58	1.18 (0.74–1.87)	0.48
				85 (0.72)	30 (0.25)	3 (0.03)			
	CNT	51 (0.13)		148 (0.77)	39 (0.20)	6 (0.03)			
PARK2 rs1801582	PD	27 (0.11)	0.73	CC	CG	GG	0.94	1.09 (0.65–1.83)	0.77
				93 (0.78)	23 (0.19)	2 (0.017)			
	CNT	60 (0.16)		155 (0.80)	35 (0.18)	3 (0.015)			
ANKK1 rs1800497	PD	103 (0.44)	0.62	CC	CT	TT	0.63	0.91 (0.65–1.26)	0.58
				41 (0.35)	51 (0.43)	26 (0.22)			
	CNT	177 (0.46)		58 (0.30)	93 (0.48)	42 (0.22)			
LRRK2 rs1994090	PD	32 (0.14)	0.62	TT	TG	GG	0.58	1.16 (0.71–1.88)	0.55
				87 (0.74)	30 (0.25)	1 (0.008)			
	CNT	46 (0.12)		150 (0.78)	40 (0.21)	3 (0.015)			
MAPT rs242562	PD	76 (0.32)	0.12	AA	AG	GG	0.29	0.75 (0.53–1.06)	0.11
				51 (0.43)	57 (0.48)	10 (0.08)			
	CNT	149 (0.39)		68 (0.35)	101 (0.52)	24 (0.12)			
RAIL/ SREBF1 rs11868035	PD	112 (0.47)	0.16	AA	AG	GG	0.39	1.29 (0.92–1.81)	0.13
				36 (0.31)	52 (0.44)	30 (0.25)			
	CNT	160 (0.41)		72 (0.37)	82 (0.43)	39 (0.20)			

*MA* minor allele frequency, *PD* Parkinson disease patients, CNT controls.

*p*^allelic^ *p* value allelic comparison, *p*^genotype^ *p* value genotype comparison, *p*^OR^ *p* value OR

^a^OR (CI 95%) additive model adjusted by sex, age, and ancestry.

Possible differences due to the Mexican population’s heterogeneity were explored by subdividing the sample into quartiles according to their percentage of Native American ancestry (Supplementary Table 2). The first group included 78 individuals with the lowest percentage (ranges from 32–52%), groups two and three were each made up of 78 individuals with intermediate ranges (52.1–56.5% and 56.6–65%, respectively), while 77 individuals with the highest Native American percentage of the sample (≤66%) were in the fourth group. We found differences between these groups in the genotype frequencies and OR estimations of SNPs rs1801133 and rs1491942 (Table 3). When comparing cases and controls, the genotypic frequency for rs1801133 was significantly different (*p* = 0.03) in the group with the lowest Native American percentage (ranges from 32 to 52%; OR 2.02 [CI 95% 1.02–4.04] *p* 0.043) in an additive model. No statistical differences were observed in any of the other three groups.

**Table 3:** Allele and genotype frequencies in PD patients and controls by percentage of Native American ancestry.

ID Marker	Group 1 (32–52%)					Group 2 (52.1–58.5%)					Group 3 (56.6–65%)					Group 4 (≥66%)				
	MA^a^ *n* (freq)	Genotype			OR [95% CI]	MA^a^ *n* (freq)	Genotype			OR [95% CI]	MA^a^ *n* (freq)	Genotype			OR [95% CI]	MA^a^ *n* (freq)	Genotype			OR [95% CI]
rs1801133		CC	CT	TT	2.02		CC	CT	TT	1.54		CC	CT	TT	1.54		CC	CT	TT	1.11
PD	31 (0.53)	9 (0.31)	9 (0.31)	11 (0.38)	[1.02–4.03]	34 (0.57)	5 (0.17)	16 (0.53)	9 (0.30)	[0.77–2.26]	40 (0.62)	5 (0.17)	14 (0.44)	13 (0.40)	[0.77–2.76]	32 (0.60)	4 (0.15)	14 (0.52)	9 (0.33)	[1.56–2.11]
CNT	38 (0.39)	17 (0.35)	26 (0.53)	6 (0.12)		40 (0.42)	16 (0.33)	24 (0.50)	8 (0.17)		48 (0.52)	12 (0.26)	20 (0.44)	14 (0.30)		57 (0.57)	10 (0.20)	23 (0.43)	17 (0.34)	
	*p* = 0.09	*p* = 0.02			*p* = 0.04	*p* = 0.07	*p* = 0.2			*p* = 0.2	*p* = 0.3	*p* = 0.5			*p* = 0.24	*p* = 0.87	*p* = 0.9			*p* = 0.75
rs1491942		CC	CG	GG	1.0		CC	CG	GG	1.5		CC	CG	GG	2.26		CC	CG	GG	2.94
PD	38 (0.66)	4 (0.14)	12 (0.41)	13 (0.45)	[0.50–1.99]	37 (0.62)	5 (0.17)	27 (0.56)	12 (0.40)	[0.75–3.04]	44 (0.68)	2 (0.07)	16 (0.50)	14 (0.43)	[1.04–4.91]	36 (0.66)	3 (0.12)	12 (0.44)	12 (0.44)	[1.38–6.23]
CNT	64 (0.65)	6 (0.12)	22 (0.45)	21 (0.43)		51 (0.53)	9 (0.19)	13 (0.43)	1 (0.38)		50 (0.54)	9 (0.19)	24 (0.52)	13 (0.28)		40 (0.40)	20 (0.40)	20 (0.40)	10 (0.20)	
	*p* = 0.99	*p* = 0.9			*p* = 0.99	*p* = 0.32	*p* = 0.4			*p* = 0.2	*p* = 0.1	*p* = 0.1			*p* = 0.04	*p* = 0.002	*p* = 0.01			*p* = 0.01

Group 1 *n* = 78 subjects; group 2 *n* = 78 subjects; group 3 *n* = 78 subjects; group 4 *n* = 77 subjects.

*PD* Parkinson disease group, *CNT* control group.

^a^MA minor allele, B additive model.

For rs1491942, significant allelic and genotypic frequency is observed in the subgroups with higher Native American ancestry. This polymorphism was estimated to be a PD risk factor in subjects with ≤56.5% Native American ancestry. In the subgroup with 56.6–65%, the estimation was (OR 2.26 [1.04–4.91] *p* 0.04), and in subjects with ≤66% Native American ancestry (OR 2.94 [1.38–6.23] *p* 0.01) in an additive model.

**Fig. 2:**
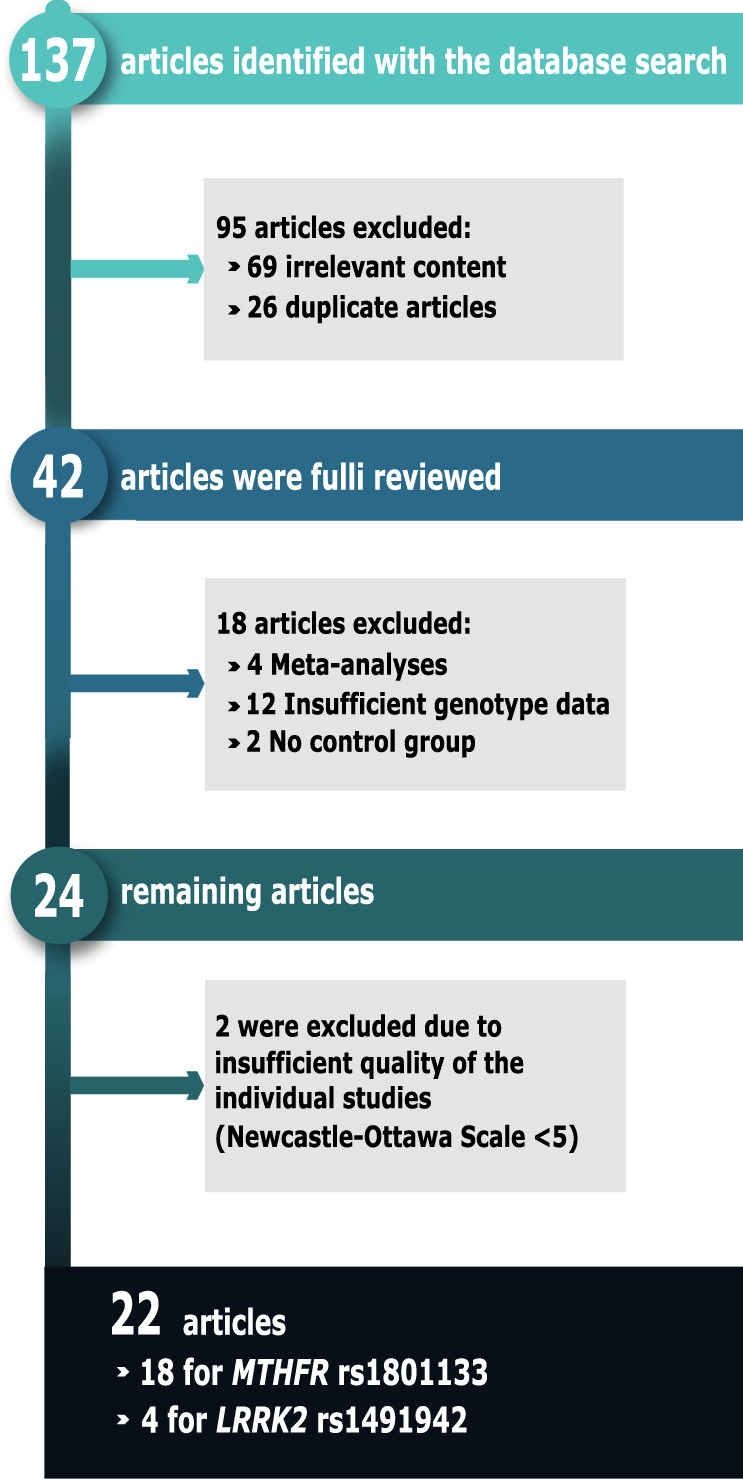
PD risk association. Flow diagram of the search and inclusion process of studies for the meta-analysis of the SNPs rs1801133 in the *MTHFR* gene and rs1491942 in the *LRRK2* gene.

Meta-analyses were performed to clarify the PD risk association of SNPs rs1801133 and rs1491942; the flow chart is shown in Fig. 2. Briefly, 137 articles were retrieved in the database search. Of these, the following were eliminated; 26 were duplicates, 69 had irrelevant content, 12 articles contained insufficient genotype data, 2 lacked control groups, and 4 were meta-analyses. After analyzing the remaining 24 papers, two more studies were ruled out due to insufficient quality (Newcastle–Ottawa Scale System Studies <5). The present study’s findings were also included in the meta-analyses.

### Meta-analysis: the association of MTHFR rs1801133 with PD risk

Nineteen studies were included in this meta-analysis (conducted on 11 European, 6 Asiatic, and 2 Mexican Mestizo populations); together, these studies comprised 2832 cases and 9074 controls. The summary characteristics of the selected studies are shown in Supplementary Table 3. No significant associations were observed for rs1801133 polymorphism and PD risk when considering an additive, dominant, or recessive model in the overall population (Table 4 and Supplementary Figs 1–3). However, in the subgroup analysis by ethnicity, there was a significant PD risk association in individuals of European ancestry under a dominant model with OR 1.17 [1.11–1.36] *p* 0.036 (Table 4 and Supplementary Figs 4–6). No significant association with PD was evident in Asian or Mexican Mestizo samples in any of the models considered (Tables 1 and 4, and Supplementary Figs 4–6). A Begg’s test detected no publication bias *p* < 0.005 (Table 4).

**Table 4:** Summary of meta-analyses.

SNP	Ethnicity	Studies	Genetic model	OR	95% CI	*p* Value	Model	*I*^2^%	Begg’s test *p* Value
rs1801133	Overall	20	T vs C	1.12	0.98–1.28	0.094	R	64.3	0.347
			TT + TC vs CC	1.14	0.96–1.36	0.111	R	53.5	0.417
			TT vs TC + CC	1.15	0.89–1.48	0.271	R	53.9	0.721
	European	12	T vs C	1.12	0.94–1.37	0.190	R	53.7	0.631
			TT + TC vs CC	1.17	1.11–1.36	0.036	F	13.8	0.537
			TT vs TC + CC	1.12	0.78–1.61	0.407	R	56.9	0.537
	Asian	6	T vs C	1.19	0.90–1.58	0.211	R	73.2	0.260
			TT + TC vs CC	1.22	0.87–1.72	0.240	R	70.4	0.260
			TT vs TC + CC	0.95	0.72–1.27	0.750	F	38.7	0.060
	Mexican Mestizos	2	T vs C	1.05	0.50–2.19	0.894	R	90.9	0.999
			TT + TC vs CC	0.95	0.32–2.72	0.926	R	87.5	0.999
			TT vs TC + CC	1.14	0.46–2.83	0.769	R	84.7	0.999
rs1491942	Overall	6	C vs G	1.25	1.10–1.14	0.012	R	81.7	0.548
	European	4	C vs G	1.14	1.07–1.22	<0.001	F	26.1	0.308
	Asian	2	C vs G	1.43	0.85–2.42	0.181	R	95.8	0.999
	Mexican Mestizos	1	C vs G	1.69	1.29–2.32	0.203	R	ND	ND

*R* random effects model, *F* fixed-effects model.

### Meta-analysis: the association of LRRK2 rs1491942 with PD risk

Five studies were included in this meta-analysis (three considered only a European population, one only Asiatic, one European and Asiatic subjects, and one Mexican Mestizo). These studies included 13,117 cases and 10,154 controls. The summary characteristics of the selected studies are shown in Supplementary Table 4. The published data were only enough to evaluate the additive model of this polymorphism. A significant association was observed between *LRRK2* rs1491942 and PD in the overall population and for the Caucasian group (Table 4, and Supplementary Figs 7 and 8). The *p* value of the Begg’s regression test revealed no publication bias *p* < 0.05 (Table 4).

## Discussion

Detection of the genetic susceptibility factors of PD has been the aim of a growing number of investigations. However, as most of these studies focus on European and Asian populations, specific populations such as Mexicans are underrepresented in these findings. Our investigation in a Mexican Mestizo population of known susceptibility factors for PD identified an association for *MTHFR* rs1801133 and *LRRK2* rs1491942 gene variants. These have previously been identified as risk variants for PD in European populations^52–55^.

The MTHFR gene located on chromosome 1p36.3 synthesizes the homodimeric cytoplasmic flavoprotein methylenetetrahydrofolate reductase. This gene is involved in the metabolism of the amino acids homocysteine and methionine, synthesis of nitrogen bases, methylation processes, and gene regulation^56–60^. While multiple polymorphisms have been described for *MTHFR*, the SNP rs1801133, also called C677T, is the most frequently investigated due to its functional impact. This polymorphism is associated with altered folate distribution, which decreases *MTHFR* enzyme activity in the catalytic region and may increase homocysteine levels^57^. In patients with PD, the C677T variant has been associated with increased homocysteine levels that precipitate damage mechanisms promoting neurodegeneration. Therefore, this variant is currently a target of PD research; however, the results have been contradictory^42,53,58,61–74^.

In our overall sample, the allelic and genotype distribution was not found to be associated with PD. However, when stratification by ancestry is performed, the risk association was observed in the group with the highest percentage of European ancestry. This information is consistent with the results of our meta-analysis and coincides with other works that describe a significant PD risk association of the rs1801133 variant in the European population^52,53^. Although verification is necessary, our findings suggest that heterogeneity in the structure of the subpopulations may explain the differences in findings for SNP rs1801133 in PD studies.

Contrary to our findings, a previous study in Mexican subjects reported the C677 allele as a PD risk factor^42^. However, the population of their study was limited to subjects from the northeastern and central regions of Mexico; therefore, geographical differences in the contribution of Native American and European ancestry in the Mexican population could explain this discrepancy, as Mexico has been shown to have an increasing north-to-south gradient of Native American ancestry^27,28,75^. However, as Garcia et al. did not report their sample’s subpopulation structure, this possibility cannot be assessed.

The *LRRK2* gene has been widely associated with pathophysiological mechanisms of both familial and sporadic PD^76^. Based on this gene’s protein sequence, several domains have been identified, such as interaction with other proteins, dimerization, GTPase, and kinase activity. These domains suggest functions in different regulatory mechanisms; cell signaling, protein complex formation, synaptic vesicle trafficking, protein recycling via retrograde trafficking pathways, autophagy regulation, among others^54,55,77^. Although mutations in this gene are present in 1–13% of PD cases, the role these variants play in the disease is still a subject of research and debate.

Concordant with previous studies^78–80^, the minor G allele for SNP rs1491942 was identified as a PD risk factor in our cohort. The same effect was observed in the genotype under dominant and recessive models. Our meta-analysis showed a PD risk association for G allele in the overall population (OR 1.25 [1.10–1.41] *p* 0.012); however, when adjusted for ethnicity, the association was conserved in Caucasian (OR 1.14 [1.07–1.22] *p* < 0.001) and Mexican populations (OR 1.69 [1.20–2.32] *p* 0.02), but not for Asians (OR 1.43 [0.84–2.42] *p* 0.18). Interestingly, subdividing our Mexican Mestizo sample by their percentage of ethnicity revealed a risk association only for the groups with >56% of Native American ascendance (Table 3). The obtained evidence suggests a risk association for rs1491942 and PD in the Mexican population with an even greater effect than previously described in populations of European descent. Although this result will need corroboration, it suggests that polymorphism rs1491942 may be useful as a risk marker of PD in Mexican Mestizos, particularly for subjects with greater Native American ancestry.

The spectrum and frequency of individual variants differ among ethnic groups and geographical locations, making comparisons across populations difficult. Our results highlight the importance of factoring the subpopulation structure into the analysis of genetic factors of PD in ethnically diverse populations. Replication studies must consider these differences when identifying and comparing PD risk factors in distinct populations.

While the moderate number of samples and polymorphisms analyzed are limitations of this work, a significant PD risk association was found for polymorphism rs1491942 in our sample of Mexican Mestizos. However, these differences were dependent on the subject’s percentage of ethnic ancestry.

Other limitations of our work include the moderate number of samples analyzed and low statistical power; replication studies are needed to corroborate these results. To our knowledge, of all published data on PD risk variants in Mexican Mestizo individuals, this is the first study to consider ancestry and includes a greater number of SNPs. Nonetheless, this work’s scope is limited; large-scale genomic studies are needed to map loci and risk variants shared with other groups and identify additional population-specific genetic variations. However, technological, economic, and ethical issues make it difficult to collect sufficient data from underrepresented groups^81^. On the other hand, the discrepancies between the replication studies conducted in different populations may be attributed to genetic and environmental diversity. While genomic studies contribute greatly to our understanding of complex diseases, they rarely integrate relevant information such as environmental factors (exposure to toxins, lifestyle habits, and nutritional aspects). These factors vary significantly between populations and have been linked to the development of various disorders, including PD^82–84^. Although case–control association studies of candidate SNPs, such as this one, continue to be a viable option for poorly studied groups with high genetic diversity, the inclusion of environmental factors to the analysis of complex traits is necessary to validate or rectify the role attributed to risk loci identified in other populations.

In summary, our case–control study found PD risk association for the polymorphisms MTHFR rs1801133 and *LRKK2* rs1491942 in the sample of Mexican Mestizo subjects. When relevant data from meta-analyses of these two SNPs and the proportion of ethnic ancestry were integrated into analysis, *MTHFR* rs1801133 was found to confer susceptibility to PD in subjects with a high percentage of European ancestry, and a more significant effect of LRKK2 rs1491942 was detected in Mexican Mestizo individuals with a high percentage of Native American ancestry. The authors consider that the association of these two SNPs and none of the other known PD-related markers derived from European and Asian cohorts merits further investigation into the functional consequences (e.g., changes in gene expression or alterations in protein levels or activity) of these shared risk factors. Identifying and studying the risk factors common to all populations will help elucidate the key biological processes of PD development.

The absence of the remaining 14 PD risk associations in our sample indicates the need for a GWAS of the Mexican population with subpopulation analysis to identify PD-associated variants that are rare in non-European populations and, therefore, not included as known genetic risk factors. Furthermore, identifying differences in LD structure around the causal variants within this population could lead to insights that shed light on the complex role of genetics in this neurological disorder.

## Methods

### Patients and controls

For the case–control study conducted between 2015 and 2017, 311 subjects were recruited from three hospitals; in the city of Durango, General Hospital 450, and Hospital Santiago Ramón y Cajal ISSSTE, and in Mexico City, General Hospital Dr. Manuel Gea González. To assure representation of Mexican Mestizos, only Spanish-speaking subjects, born in Mexico with Mexican ascendancy (at least parents and grandparents), were considered. The cohort included 118 patients (60 males and 58 females, mean age 69 ± 10 years) diagnosed with PD by an experienced neurologist according to the UK Parkinson’s Disease Society Brain Bank clinical diagnostic criteria, but with no familial history of the disease. The control group consisted of 193 unrelated individuals age- and sex-matched with the PD patients (96 males and 97 females mean age 69 ± 8 years), with no PD diagnosis or a personal or familial history of neurodegenerative diseases.

The subjects’ demographic characteristics, clinical data, and lifestyle were recorded. Their cognitive condition was evaluated with the Mini-Mental State Exam and depression with the Hamilton Depression Rating Scale. The UPDRS score and HY scale were used for determining PD severity.

The internal Ethics and Research Committees of the participating hospitals (no. 49-21-2015/no. Eel-56-2013) approved this study, and it was carried out per the Declaration of Helsinki’s ethical principles for medical research involving human subjects developed by the World Medical Association. All participants gave written informed consent.

Peripheral blood samples were collected from all the subjects. Genomic DNA was isolated from whole blood using a QIAamp DNA extraction Kit (Qiagen, Hilden, Germany). DNA purity and concentration were determined spectrophotometrically, and the samples were stored at −80 °C until use. For biochemical determinations, the blood samples were centrifuged at 3000 r.p.m. for 15 min. The serum and total cholesterol, uric acid, and glucose levels were then quantified using the Random Access Automatic Biochemical Analyzer for Clinical Chemistry and Turbidimetry A15 (BioSystems S.A.).

### Selection of single-nucleotide polymorphisms and genotyping

For the selection of variants, a search was performed in the Pubmed database, considering articles published up to September 2014 using the keywords: “Parkinson’s disease,” “Polymorphism, Single Nucleotide,” or/and “Genetic Association Studies,” or/and “Genome-wide Association Study.”

For the final selection, preference was given to variants with a reported contribution to PD’s pathophysiological mechanisms or a PD association identified by GWAS studies (see Supplementary Table 5 for the selected SNPs’ characteristics). The 21 SNPs selected for genotyping were all associated with PD risk in more than one previous study.

Nine of these variants were linked to PD in at least two independent, unrelated cohort studies. They comprise: rs13312, a variant in the 3′-untranslated region of the ubiquitin-specific protease 24 gene (*USP24*), associated with (PARK10)^85,86^, a susceptibility locus for PD; rs3766606, an intronic modification in the deglycase gene (*PARK7*), linked with parkinsonism in Chinese and European populations^87,88^; rs1801474 and rs1801582, two missense variants in the parkin RBR E3 ubiquitin-protein ligase gene *(PRKN*), associated with PD risk^89–91^; rs1800497, often correlated with neurological disorders, including PD, is located in the coding region of the ankyrin repeat and kinase domain containing the gene (*ANKK1*), which controls dopamine synthesis in the brain^50,92,93^; rs1801133, in the methylenetetrahydrofolate reductase gene (*MTHFR*), possibly implicated in PD^61,65^; rs334558, a polymorphism in the glycogen synthase kinase 3 beta gene (*GSK3B*) potentially a protective factor for PD in Asian populations^94,95^; rs6280, in the dopamine receptor D3 gene (*DRD3*), implicated in both PD vulnerability and motor complications^96,97^; and rs242562, a polymorphism in the microtubule-associated protein tau gene (*MAPT*), associated with PD^98^.

The 12 additional variants were selected because of their PD association reported in complete genome studies (GWAS). These include: rs823128, rs823156, and rs947211, three variants vinculated with the susceptibility locus *PARK16* in Asian and European populations^99,100^; rs2736990, rs356220, and rs356219, polymorphisms in the synuclein alpha gene (*SNCA*), proposed as PD risk factors in Asian and European populations^100–103^; rs1491942, rs33949390, and rs34778348, variants in the LRRK2 gene, frequently associated with PD worldwide^104^; and the variants, rs6812193 in *FAM47E*, associated with a significant risk of developing PD; and rs11868035 in *SREBF1* (refs. ^78,104^).

Genotyping was carried out using predesigned TaqMan SNP Genotyping Assays (by Applied Biosystem CA) with the following assay ids, SNP id and gene name (C_7516392_10, rs1491942; C__63497592_10, rs33949390; C__63498855_10, rs34778348 C_1867882_10, rs1994090 *LRRK2*; C_1020193_10, rs356219; C_3208948_10 rs2736990; C_1020192_10, rs356220 *SNCA*, C_8701299_10, rs1801582; C_8947865_10, rs1801474 *PRKN*; C_2966873_10, rs3766606 PARK7; C_998739_10, rs13312 USP24; C_31139749_10, rs6812193 *FAM47E*; C_949770_10, rs6280, *DRD3*; C_3224431_10, rs1800497,*ANKK1*; C_3202957_10, rs242562, *MAPT*; C_375742_10, rs823156; C_8721272_10, rs947211, *SLC41A1*; C_1202883_20, rs1801133 *MTHFR*; C_31463202_10, rs11868035 *SREBF1*; C_11451241_10, rs823128 *NUCKS1*; and C_905680_10, rs334558 *GSK3B*).

The real-time polymerase chain reaction (PCR) with allelic discrimination analysis was performed according to the standard protocol. Briefly, 10 ng of genomic DNA mixed with 0.625 µL of Taqman SNP genotyping assay and 5 µL of Universal PCR Master Mix (Applied Biosystem CA) adjusted with nuclease-free water for a final volume of 20 µL per well. The mix was added to a 48-well plate and amplified 40 cycles in a StepOne machine (Applied Biosystems, Foster City, CA USA). All subjects were genotyped; 10% of the assays were randomly selected for replication, and these tests were all consistent with our initial results.

A validated panel of 32 AIMs designed for Mexican individuals was used for the stratification correction and estimation of global ancestry^51^. The SNP genotyping assays were generated with the OpenArray® platform by Quantstudio™ (Applied Biosystem, CA), per the manufacturer’s recommendations.

The comparative analysis was performed using ADMIXTURE software set at *k* = 2 to discriminate between European and Native American ancestries. A dataset of 95 non-related individuals from the European Utah population (CEU) plus 38 individuals of Mayan or Zapoteca origin was included to represent the parental populations^51^.

### Statistical analysis

Since study participants were recruited from both Northeastern Mexico and Mexico City, differences in the demographic and clinical characteristics potentially attributable to the place of recruitment were compared using the Mann–Whitney test for continuous variables and Fisher’s exact test for the categorical variables. When comparing differences in demographics and clinical characteristics between PD cases and controls, either a Student’s *t* test or Mann–Whitney test was used depending on the distribution of the continuous variables. Chi-square and Fisher’s exact tests were used to assess differences between groups for categorical variables. For association analysis of the SNPs, we defined the ancestral alleles as the major allele, i.e., higher in frequency (according to the National Center for Biotechnology Information SNP database). The HWE of the control group was verified with a chi-square test. Linkage disequilibrium was examined using Haploview software (Broad Institute, Cambridge, MA, USA) and genotyping data from the 1000 Genomes project. Differences in genotyping and allelic frequency distribution between cases and controls were compared, using the Fisher exact test. Each SNP’s association with PD was evaluated using logistic regression models adjusted for age, sex, and percentage of Native American ancestry. OR and 95% CI were calculated for the associations, and *p* values reported. To counteract the problem of multiple comparisons, the Bonferroni correction was used to test the “universal null hypothesis”, i.e., that all tests are not significant. The threshold for statistical significance after this correction was *p* < 0.003.

To determine whether the subpopulation structure factored into the incidence of PD-associated SNPs, the differences were calculated considering each subject’s percentage of Native American ancestry. The sample was divided into quartiles. Group 1 had the lowest percentage of Native American ancestry ranging from 32 to 52%, group 2 contained 52.1–58.5%, group 3 from 56.6 to 65%, and group 4 had the highest percentage ≥66%. The association with PD was evaluated for these groups using stratified logistic regression models adjusted for age and sex; *p* values <0.05 were considered statistically significant.

Furthermore, meta-analyses were carried out for the SNPs shown to have a significant association with PD. The following search criteria were used to identify related studies in the PUBMED and ScienceDirect databases: papers published before July 2019 using the keywords: Parkinson’s disease (PD) and SNP (or polymorphism or mutation or variant) rs1801133 (or C677T or Ala222Val and MTHFR) and/or rs1491942 (and *LRRK2*). In addition, potentially relevant literature was identified from the reference section of related studies. The following selection criteria were used: (1) human case–control design, (2) evaluation of genetic susceptibility to PD, (3) OR reported with 95% confidence interval, or enough data to estimate the OR, and (4) English language publication. Exclusion criteria were: (1) duplicate studies, (2) animal studies, case reports, and conference abstracts, (3) only familial PD research, and (4) evaluation of the associations between SNPs and PD therapy response or prognosis. Also, the selected studies’ quality was assessed using the Newcastle–Ottawa Scale^105^. Studies were scored independently by two reviewers, and articles with scores <5 were discarded. The associations between polymorphism rs1491942 in the *LRRK2* gene and PD susceptibility were estimated based on pooled ORs and 95% CI. The *p* value of Cochran’s *Q* statistic was evaluated to determine heterogeneity. If *p* < 0.10 or *I*² > 50, a random effects model was used; in the absence of heterogeneity, a fixed-effects model was used. The *Z* test was used to determine if the OR was significant, and a *p* value <0.05 was considered statistically significant. Publication bias was determined using a Begg’s linear regression test; a *p* value <0.05 was considered evidence of bias. A sensitivity analysis was also performed. The STATA software (version 13.0; STATA Corporation, USA) was used for all specified statistical analyzes.

## Supplementary information

Supplementary Material

nr-reporting-summary

## Data Availability

The data that support the findings of this study are available from the corresponding author upon reasonable request.
