# Supplementary material for: Differences in MTHFR and LRRK2 variant’s association with sporadic Parkinson’s disease in Mexican Mestizos correlated to Native American ancestry
Source: NPJ Parkinsons Dis. 2021 Feb 11;7:13. doi: 10.1038/s41531-021-00157-y (PMC7878860; doi:10.1038/s41531-021-00157-y)
Supplement: Supplementary file 1 — Supplementary Material [file 41531_2021_157_MOESM1_ESM.pdf]

Supplementary Material

Supplementary Tables

Table 1. Clinical and demographic characteristic of the study population						
	Cases			Controls		
	Northeastern Mexico (n= 75)	Central Mexico (n=43)	p	Northeastern Mexico (n=92)	Central Mexico (n=101)	p
Sex ratio: Males n(%)	38 (50)	22 (51)	0.99[2]	44 (47)	53 (52)	0.15[2]
Average age at enrollment (range)	70.77 [62-79]	68.44 [63-72]	0.18[1]	71.1 [64-77.5]	68.61 [63-73]	0.07[1]
BMI, kg/m^2	27.83 [24 -31]	26.60 [24-29]	0.43[1]	27.03 [23-30]	27.33 [24-30]	0.33[1]
Total cholesterol, mg/dl	174 [133-207]	179[153-206]	0.60[1]	188 [167-215]	202 [172-226]	0.06[1]
Glucose, mg/dl	104 [83-105]	99.12[91-103]	0.21[1]	108 [86-107]	112 [94-114]	0.002[1]
Uric acid, mg/dl	5.17 [4.4-5.9]	5.33 [4-6.39]	0.25[1]	5.93 [5.0-6.9]	6.12 [5.1.7.1]	0.45[1]
Cognitive impairment n(%) (by MMSE-test)	26 (34%)	11 (25%)	0.40[2]	20 (21%)	31 (30%)	0.19[2]
Age at onset, years	63.9 [55-72]	64.4 [58-69]	0.84[1]			
UPDRS total score	74 [44-92.5]	68.4 [39-88]	0.46[1]			
UPDRS III score	40.6 [23-5]	40.1 [20-53.5]	0.88[1]			
HY scale	2.7 [2-3]	2.7 [2-3]	0.84[1]			

Shows mean values (Interquartile range) and frequency (%). p values were determined with U Mann-Whitney[1] and Fisher's exact[2] tests. BMI: Body Mass Index HAM-D: Hamilton Depression Rating Scale, MMSE: Minimental state exam, UPDRS: Unified Parkinson's Disease Rating Scale total score and score Part III — Motor Examination, HY =Hoehn and Yahr scale

Table 2. Odds ratio estimates by percentage of Native American ancestry for remaining non-significant SNPs								
Gene ID marker	Group1 (32-52%)	p	Group 2 (52.1 - 58.5%)	p	Group 3 (56.6 – 65%)	p	Group 4 (≥66%)	p
USP24 rs13312	0.54[0.15-1.95]	0.35	3.25[0.15-1.93]	0.40	0.49[0.10-2.18]	0.31	0.93[0.27-3.23]	0.92
PARK7 rs3766606	1.49[0.58-3.84]	0.41	0.39[0.08-1.82]	0.23	0.68[0.20-2.26]	0.54	0.69[0.16-2.97]	0.63
NUCKS rs823128	0.48[0.14-1.59]	0.23	0.79[0.29-1.71]	0.35	1.05[0.38-2.86]	0.92	1.40[0.64-3.05]	0.4
SLC41A1 rs823156	0.49[0.20-1.17]	0.11	0.71[0.29-1.71]	0.50	1.32[0.64-2.72]	0.44	0.74[0.34-1.60]	0.45
DRD3 rs6280	0.90[0.96-1.78]	0.77	0.87[0.44-1.72]	0.70	0.75[0.40-1.41]	0.38	0.70[0.35-1.41]	0.33
GSK3B rs334558	0.96[0.48-1.96]	0.92	1.53[0.80-2.92]	0.20	0.65[0.31-1.36]	0.26	1.62[0.76-3.47]	0.22
FAM47E rs6812193	1.20[0.60-2.38]	0.60	1.29[0.58- 2.88]	0.52	0.86[0.36-2.01]	0.73	1.62[0.60-4.32]	0.33
SNCA rs356219	0.7[0.36-1.38]	0.31	0.84[0.44-1.59]	0.60	0.71[0.35-1.47]	0.37	1.04[0.50-2.17]	0.9
PARK2 rs1801474	1.44[0.54-3.78]	0.47	1.32[0.54-3.24]	0.53	0.75[0.29-1.91]	0.55	0.85[0.32-2.05]	0.73
PARK2 rs1801582	1.43[0.54-3.78]	0.46	0.94[0.36-2.46]	0.91	0.97[0.37-2.53]	0.96	0.90[0.20-4.04]	0.94
ANKK1 rs1800497	1.52[0.74-3.14]	0.25	1.52[0.39-1.56]	0.41	0.88[0.48-1.63]	0.70	0.72[0.38-1.37]	0.32
SLC2A13rs1994090	0.99[0.33-2.93]	0.99	0.68[0.25- 1.83]	0.45	1.47[0.54-4.12]	0.46	2.26[0.82-6.24]	0.33
SREBF1rs11868035	0.80[0.39-1.64]	0.55	0.57[0.19-1.69]	0.32	2.32[0.82-6.53]	0.11	0.84[0.27-2.58]	0.13
MAPT rs242562	0.88[0.44-1.73]	0.71	0.80[0.34-1.87]	0.62	0.58[0.27-1.23]	0.16	0.55[0.26-1.18]	0.11
OR estimation using an additive model, adjusted by sex and age. Group 1 n=78 subjects with 32 - 52% Native American ancestry; Group 2 n=78 subjects with 52.1% - 56.5% Native American ancestry; Group 3 n=78 subjects with 56.6 - 65% Native American ancestry; Group 4 n=77 subjects with ≤66% Native American ancestry.								

Table 3. Summary characteristics of studies included in the meta-analysis of MTHFR rs1801133 and PD risk										
				PD			Controls			HWE
Author	Year	Country	Ethnicity	n	Gender (% Male)	Age	n	Gender (% Male)	Age	
De Lau et al.	2005	Netherlands	European	65	46%	74.9±7.2	5855	32%	68.7±8.6	0.21
Wullner et al.	2005	Germany	European	342	71%	59.9±9.0	342	50%	58±16	nd
Religa et al.	2006	Poland	European	114	nd	69.9	100	nd	71.2	1
Rodriguez-Oroz et al.	2006	Spain	European	77	58%	69.9±6.5	28	51.70%	68.5 ±3	0.72
Todrovic et al.	2006	Serbia	European	113	56%	61.1±9.0	53	64%	60.8±13.1	0.78
Caccamo et al.	2007	Italy	European	49	45%	64.2±7.5	86	47%	64.1±7.1	0.6
Dorszewska et al.	2007	Poland	European	98	62%	60.8±10.7	50	32%	44.6±16.2	0.71
Camicioli et al.	2009	Canada	European	51	59%	71.5±4.7	49	58%	71.6±4.9	0.78
Bialecka et al.	2012	Poland	European	320	51%	64.4±10.1	254	54%	64.8±9.6	0.88
Gorgone et al.	2012	Italy	European	60	45%	64.5±7.7	82	45%	64.1±7.2	0.49
Zahra et al.	2016	Malta	European	151	62%	72.9	311	55.70%	74.3	0.38
Yasui et al.	2000	Japan	Asian	90	34%	64.8±12.6	53	26%	67.8±12.2	0.25
Lin et al.	2007	Taiwan	Asian	94	45%	69.5± 7.51	146	47%	68.5± 6.1	0.6
Kumudini et al.	2008	India	Asian	151	nd	nd	416	nd	nd	0.72
Yuan et al.	2009	Taiwan	Asian	76	47%	71.3±9.8	110	44%	69.9±8.4	0.61
Fong et al.	2010	Taiwan	Asian	211	48%	71.2±8.4	218	47%	71.0±6.4	0.47
Yaun et al .	2015	China	Asian	512	60%	65.8±10.3	512	60%	65.9±10.5	0.41
Garcia et al.	2015	Mexico	Mexican mestizos	140	68%	65.46 ±11.5	216	65%	63.68± 8.8	0.13
Present study	2019	Mexico	Mexican mestizos	118	51%	69.92± 10.	193	50%	69.80±8.63	0.63

Table 4. Summary characteristics of studies included in the meta-analysis of LRRK2 rs1441942 and PD risk

Author	Year	Country	Ethnicity	PD			Controls			HWE
				n	Gender (% Male)	Age	n	Gender (% Male)	Age	
Pihlstrom et al.	2013	Norway, Sweden	European	1380	60%	68[32-90]	1295	52%	66[49-91]	ND
Soto Ortolaza et al.	2013	Poland, Ireland, United States	European	1381	61%	65.9±12.0	1328	42%	64±17	ND
Fang et al.	2016	China	Asian	583	45%	65.1±8.9	553	48.3%	65.37 ±9.03	0.67
Wang et al.	2017	Asian cohort	Asian	1940	58%	63	3273	58%	63	0.99
Wang et al.	2017	European cohort	European	7715	46%	64	3512	25%	54	0.63
Present study	2019	Mexican	Mexican mestizos	118	50.8%	69.92±10	193	50.2	69.80± 8.63	0.65

Table 5. Summary characteristics of included SNPs

No	ID SNP	Chr	Position in (bp)	Gene	Gene classification	Variant type	Alleles (minor/major)
1	rs1801133	1	11796321	MTHFR	Metabolism	missense	T/C
2	rs13312	1	55067069	UPS24	Ubiquitin specific peptidases	3'-UTR	G/C
3	rs3766606	1	7962137	PARK7	PD associated gene	intron	T/G
4	rs823128	1	205744250	NUCKS1	Transcription regulation gene	intron	G/A
5	rs823156	1	205795512	SLC41A1	Membrane trafficking and solute carriers	intron	G/A
6	rs334558	3	120094435	GSK3B	Proteins with kinase activity	5´UTR	A/G
7	rs6280	3	114171968	DRD3	Neurotransmitter receptors in signal transduction pathways	missense	C/T
8	rs6812193	4	77198986	FAM47E/SCARB2	PD risk factors identified by a GWAS	intron	T/C
9	rs356219	4	89716450	SNCA	PD associated gene	intron	A/G
10	rs1801582	6	161386823	PARK2	PD associated gene	missense	G/C
11	rs1801474	6	162201165	PARK2	PD associated gene	missense	T/C
12	rs1800497	11	113400106	ANKK1	Neurotransmitter receptors in signal transduction pathways	missense	A/G
13	rs1491942	12	40227006	LRRK2	PD associated gene	intron	G/C
14	rs1994090	12	40034759	LRRK2	PD associated gene	intron	G/T
15	rs242562	17	45949373	MAPT	Membrane trafficking and solute carriers	intron	G/A
16	rs11868035	17	17811787	RAIL/SREBF1	PD risk factors identified by a GWAS	intron	G/A

ID SNP= Identifier of Single Nucleotide Polymorphism, Chr = Chromosome; Variant types: 3'-UTR = 3'-untranslated region; 5'-UTR = 5'-untranslated region

Supplementary Figures

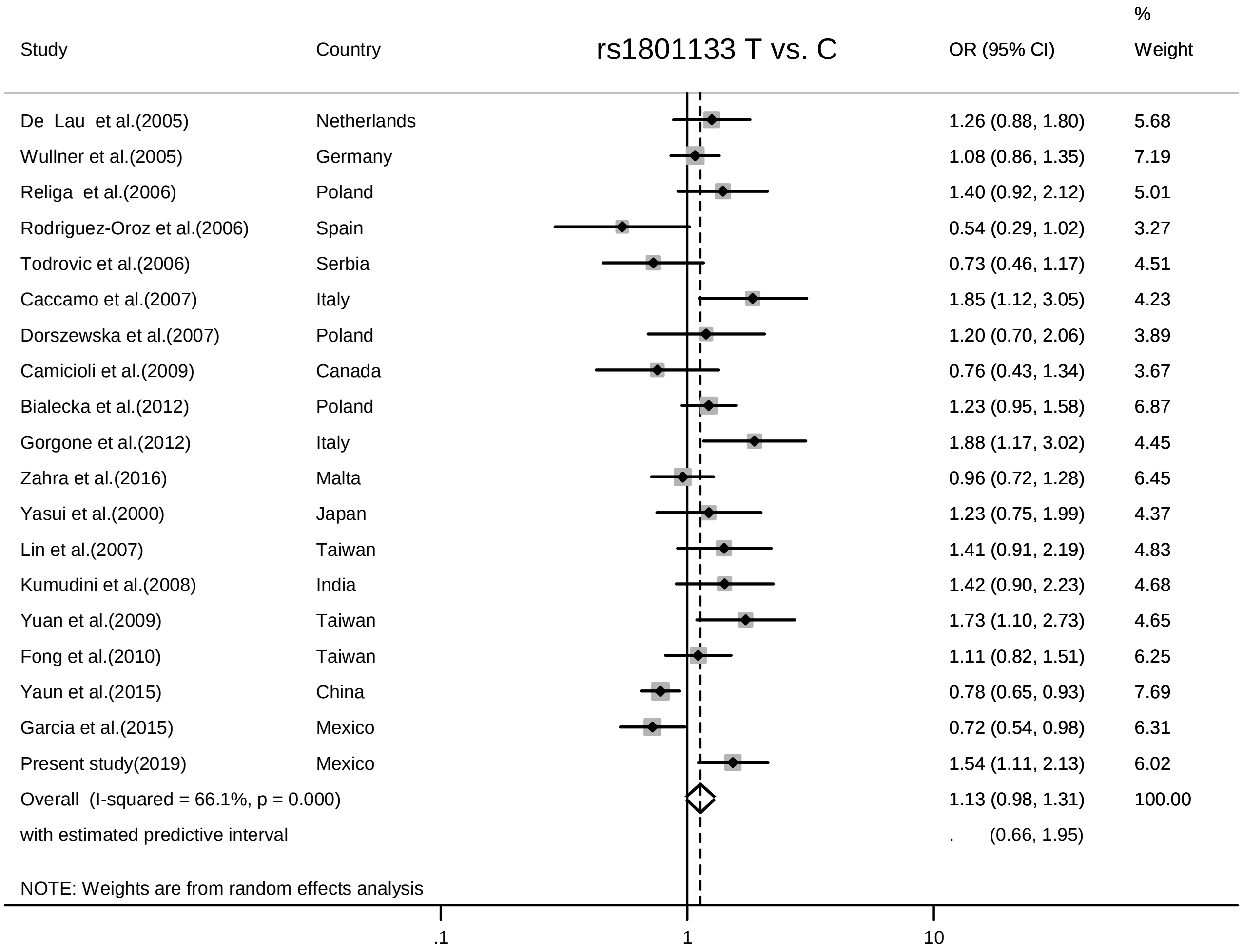

Figure 1. Forest plots from the meta-analysis of MTHFR rs1801133 allele's PD-risk association in the combined sample. "OR" odds ratio.

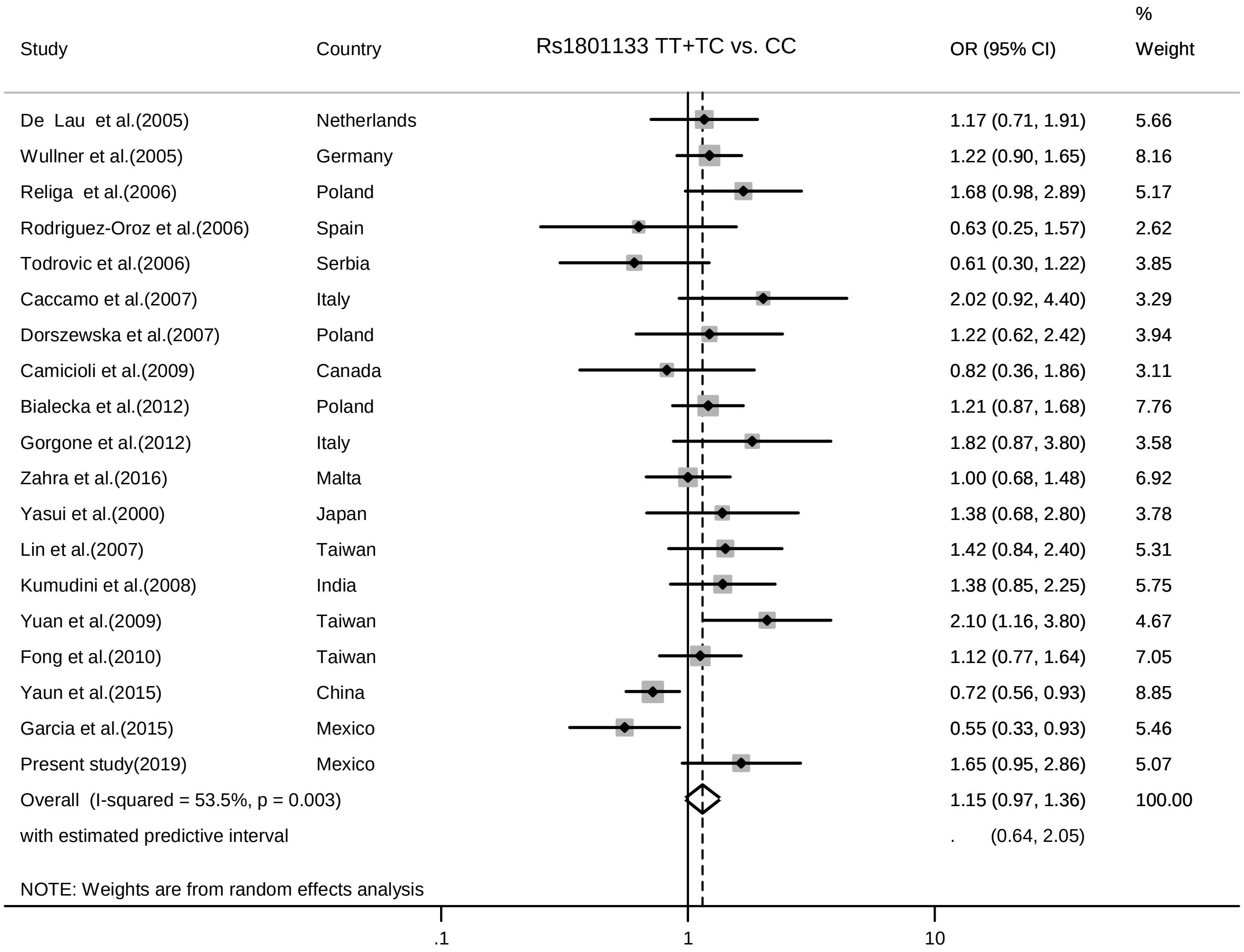

Figure 2. Forest plots from the meta-analysis of MTHFR rs1801133 (TT+TC vs. CC) genotypes' PD-risk association in the combined sample. "OR" odds ratio

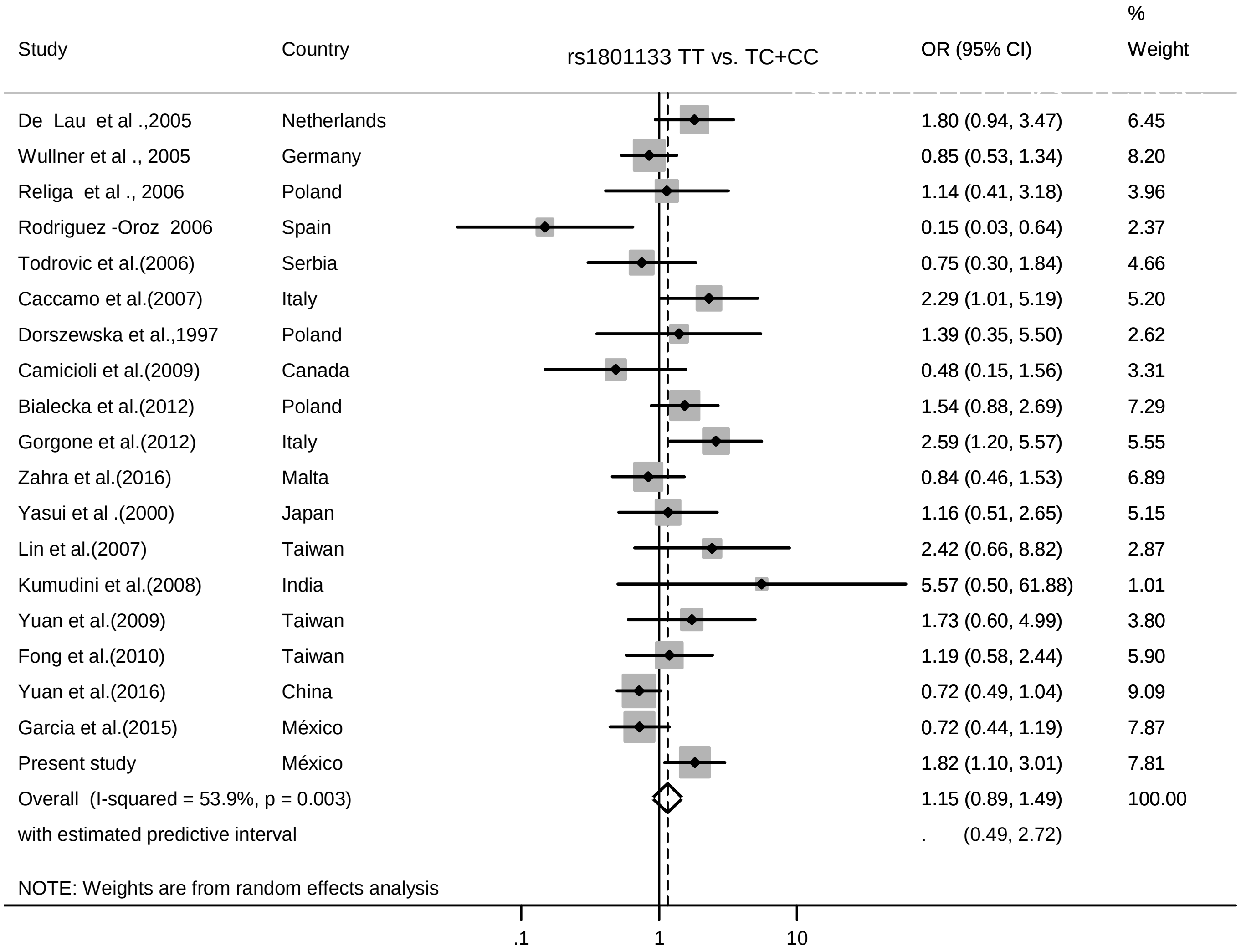

Figure 3. Forest plots from the meta-analysis of MTHFR rs1801133 (TT vs. TC+ CC) genotypes’ PD-risk association in the PD subgroup. “OR” odds ratio

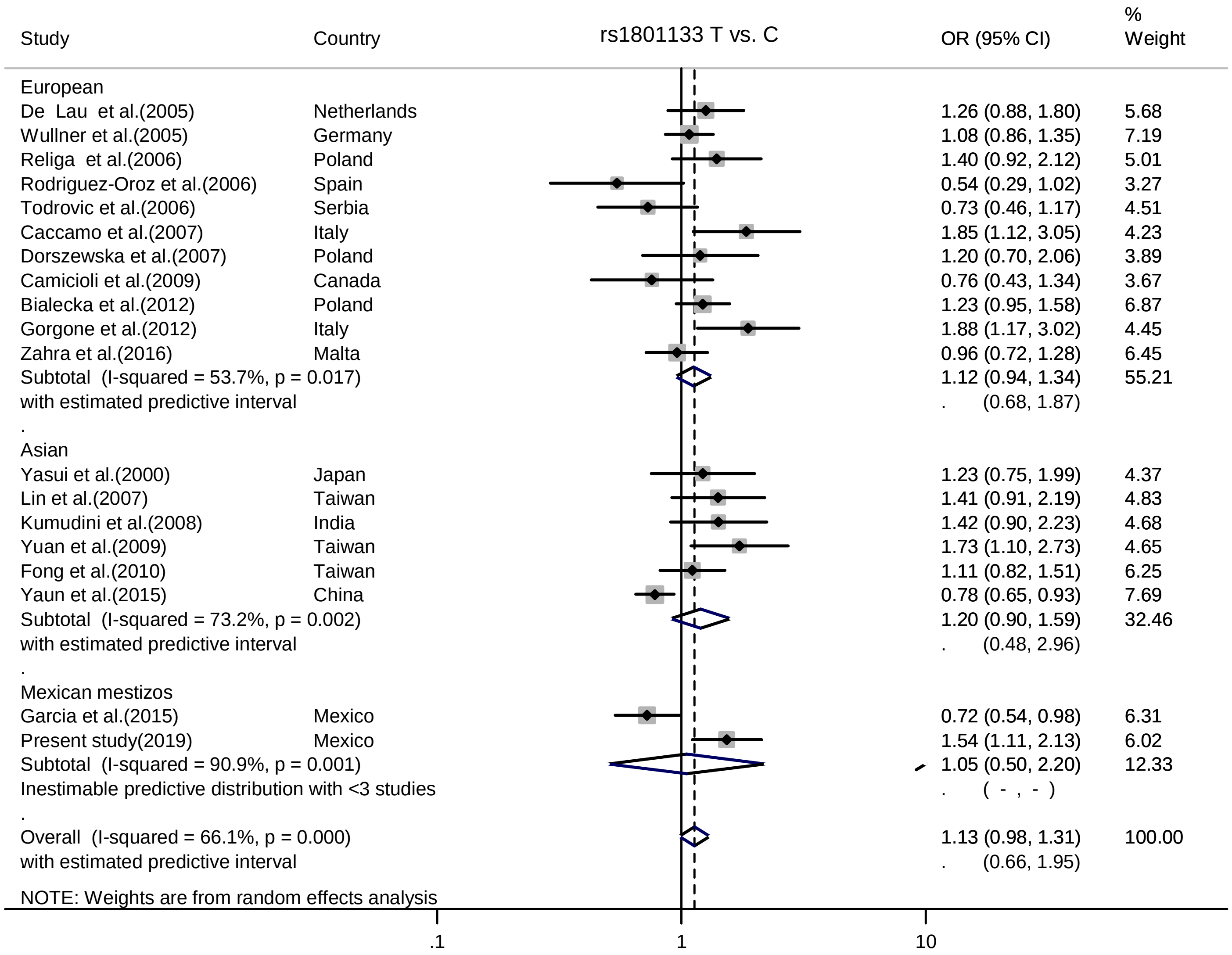

Figure 4. Forest plot from the meta-analysis of MTHFR rs1801133 (T vs. C)'s PD risk association in the PD subgroup stratified by ethnicity. "OR" odds ratio.

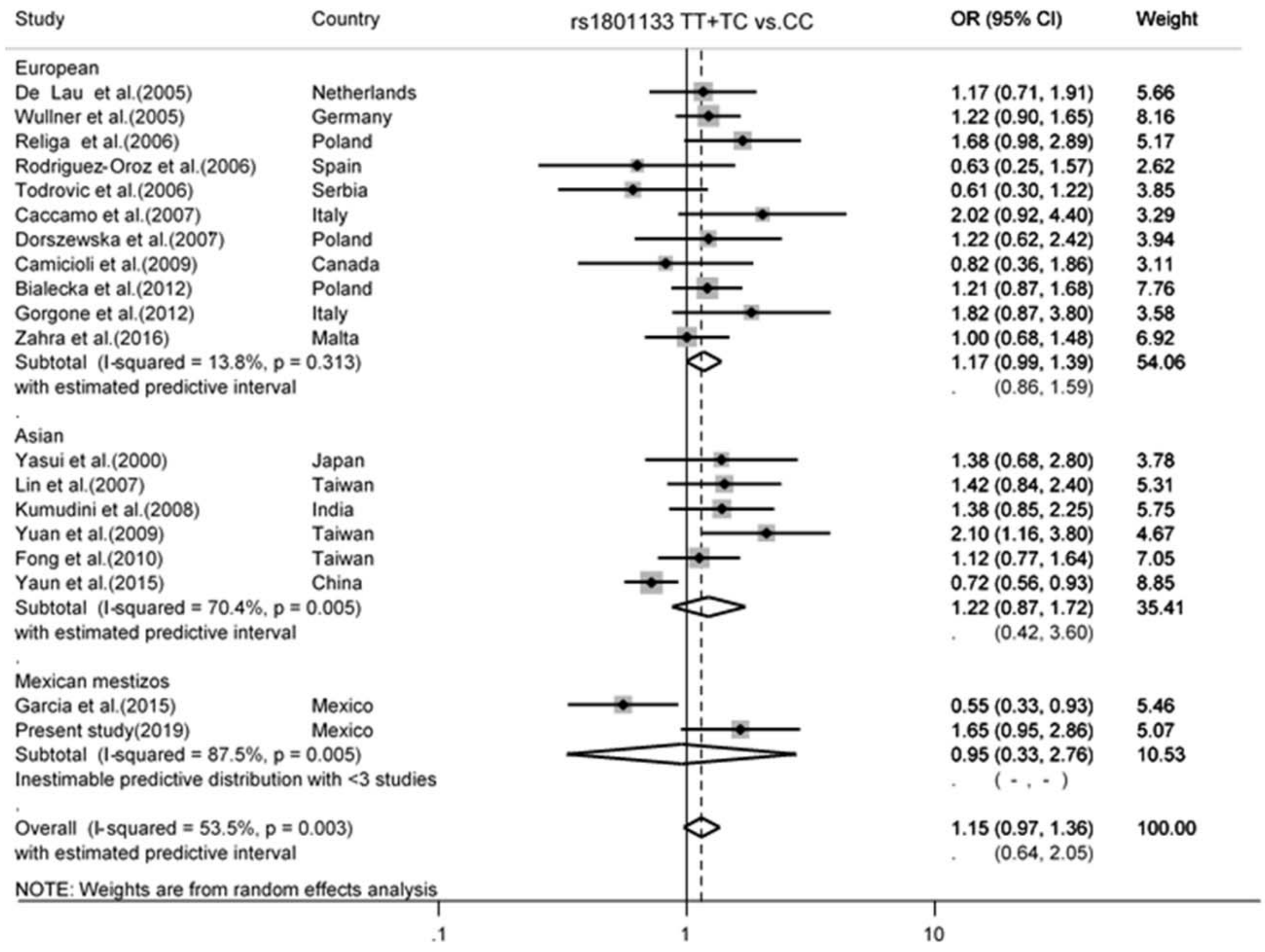

Figure 5. Forest plots from the meta-analysis of MTHFR rs1801133 (TT+TC vs. CC) genotypes' PD-risk association in the PD subgroup stratified by ethnicity. "OR" odds

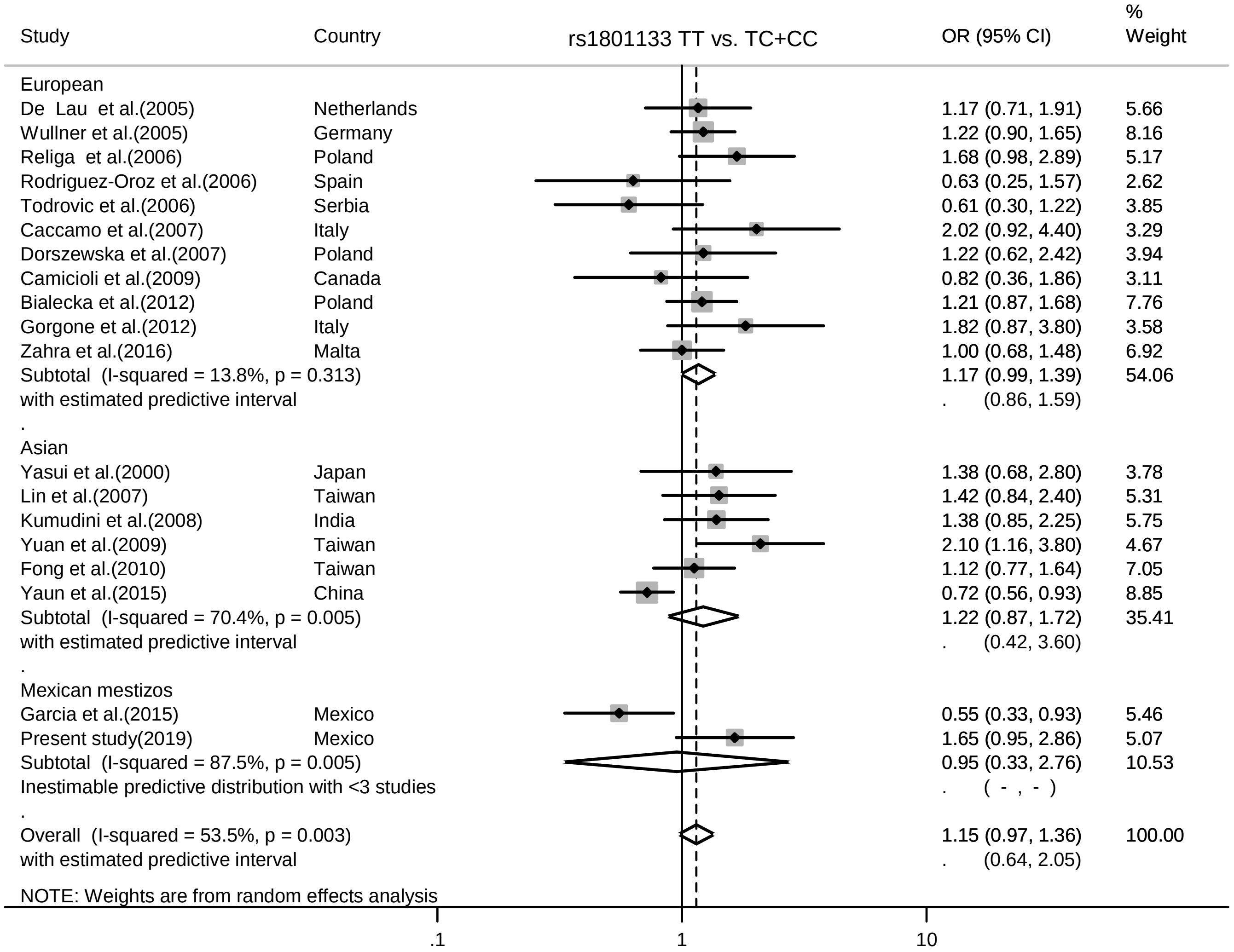

Figure 6. Forest plots from the meta-analysis of MTHFR rs1801133 (TT vs. TC + CC) genotypes' PD-risk association in the PD subgroup stratified by ethnicity. "OR" odds ratio

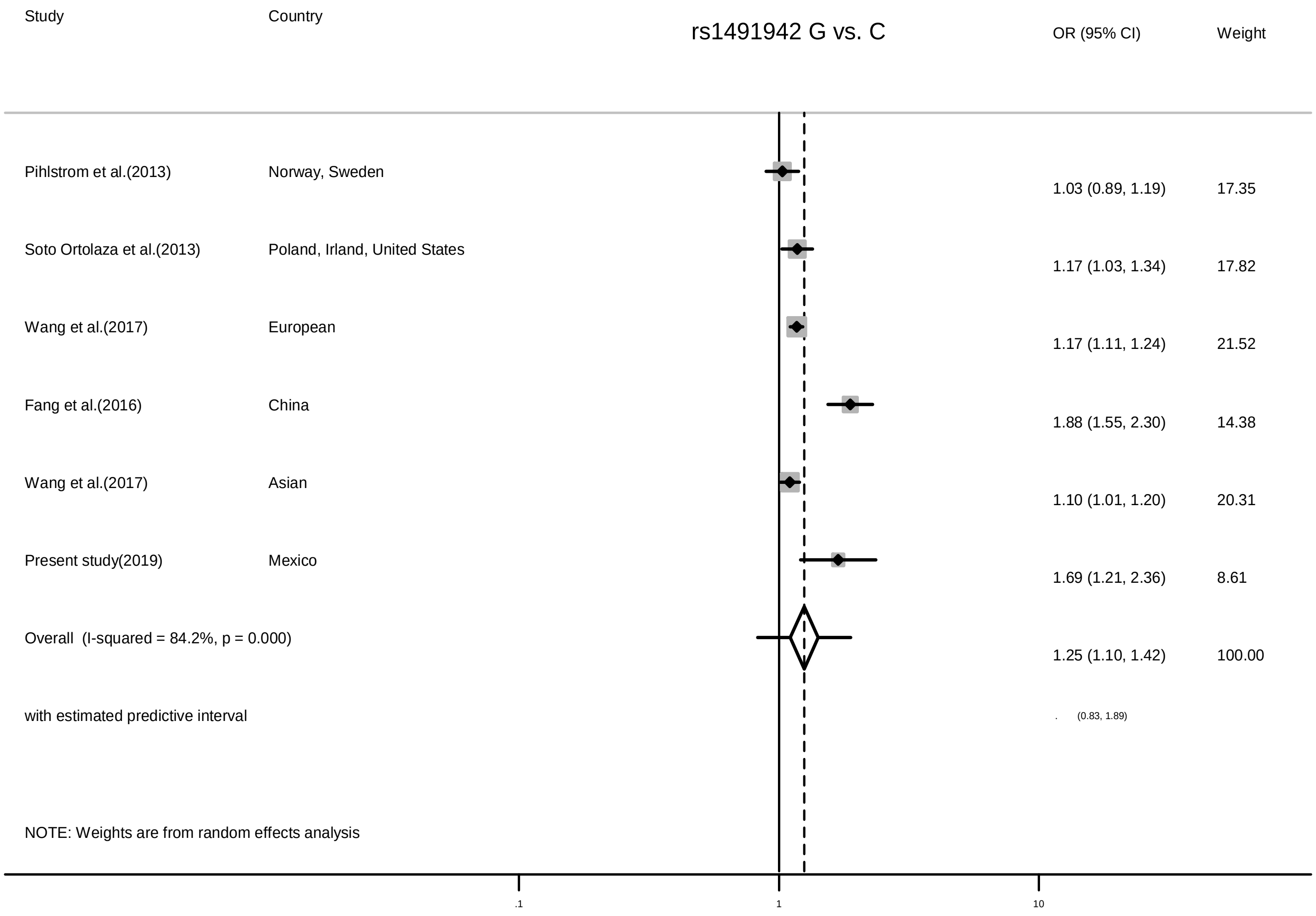

Figure 7. Forest plots from the meta-analysis of LRRK2 rs1491942 (G vs. C) allele’s PD risk association in the combined sample. “OR” odds ratio

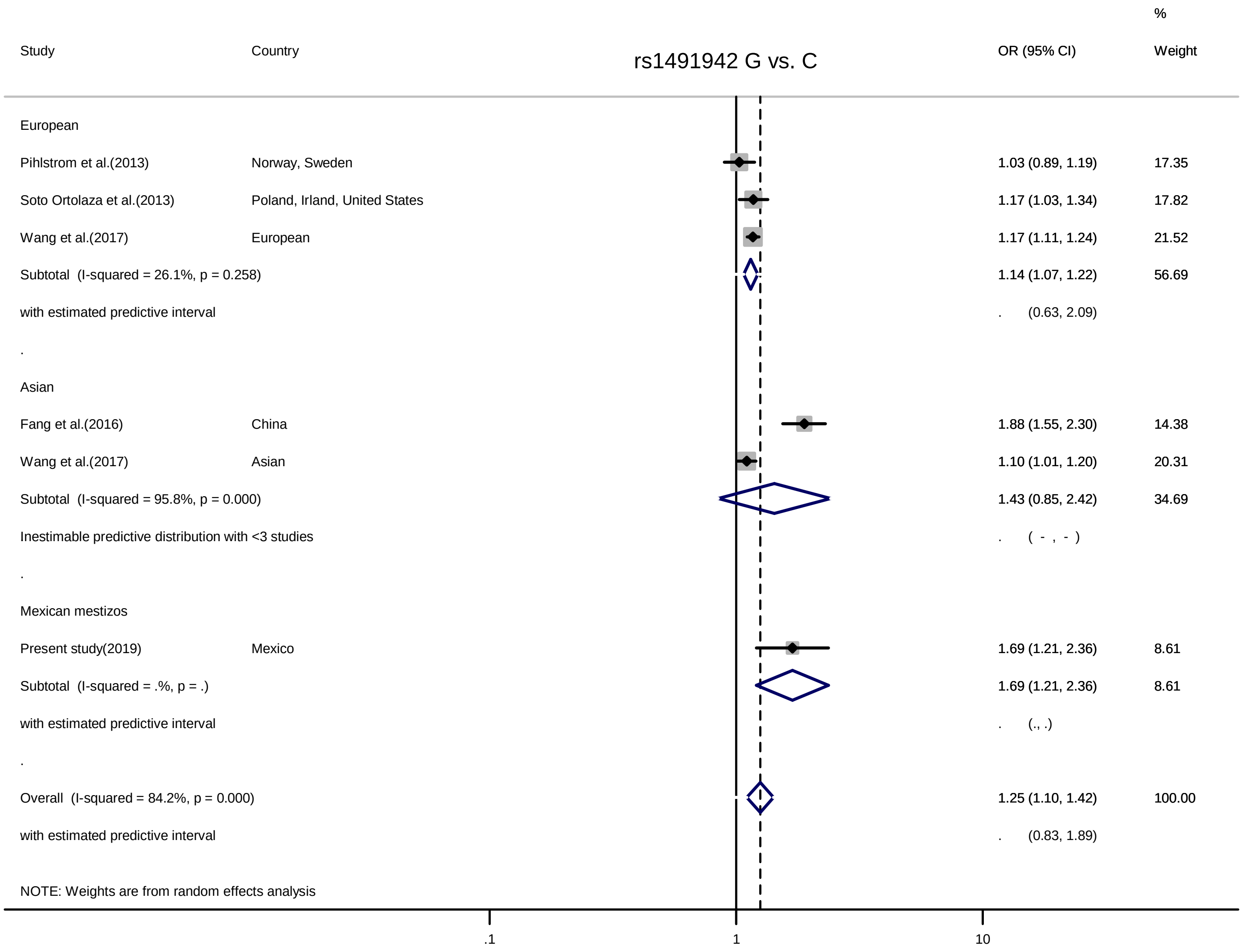

Figure 8. Forest plots from the meta-analysis of LRRK2 rs1491942 allele's PD risk association in the PD subgroup stratified by ethnicity. "OR" odds ratio
